# Obinutuzumab for treatment of membranous nephropathy in patients positive and negative for the phospholipase A_2_ receptor antibody: case reports

**DOI:** 10.3389/fimmu.2025.1561638

**Published:** 2025-04-15

**Authors:** Jinmeng Wang, Wenfeng Wu, Huiyi Wu

**Affiliations:** ^1^ Clinical Pharmacy Center, Nanfang Hospital, Southern Medical University, Guangzhou, China; ^2^ Pharmacy Department, Shandong First Medical University Affiliated People’s Hospital, Jinan, China

**Keywords:** membranous nephropathy, PLA2R, obinutuzumab, rituximab, nephrotic syndrome

## Abstract

**Introduction:**

Membranous nephropathy (MN) is a common cause of nephrotic syndrome (NS) in adults. Without treatment, two-thirds of patients with MN develop nonprogressive chronic kidney disease or end-stage renal disease within 10 years. Obinutuzumab (OBZ), which binds to CD20 and leads to a pronounced depletion of B cells, may elicit a better therapeutic response in patients with refractory MN who do not respond to rituximab or who have recurrent episodes.

**Methods:**

We describe two MN patients, one positive and one negative for the M-type phospholipase A_2_ receptor antibody (PLA_2_R Ab). Both patients had poor responses to rituximab, but had different responses to OBZ.

**Results:**

The patient positive for PLA_2_R Ab had an immunologic response, and the patient negative for PLA_2_R Ab had a nearcomplete clinical response.

**Discussion:**

OBZ appears to be a suitable alternative when other treatment options are ineffective or contraindicated. The efficacy of OBZ for treatment of refractory MN needs to be verified by large-scale multicenter clinical trials.

## Introduction

Membranous nephropathy (MN) is an autoimmune disease characterized by thickening of the glomerular capillary walls due to the deposition of immune complexes, and is the most common pathological cause of nephrotic syndrome (NS) in adults (1). About 75 to 80% of patients with primary MN have serum antibodies (Abs) against the phospholipase A_2_ receptor (PLA_2_R), although renal biopsy is the gold standard for diagnosis. The serum level of the PLA_2_R Abs can predict response to treatment (2). Cyclophosphamide (CYC) has long been the standard treatment for MN because it can prevent the development of advanced renal failure, but this drug increases the risk of malignant tumors and has potentially irreversible reproductive toxicity (3). Treatment with a calcineurin inhibitor (CNI), such as cyclosporine (CSP) or tacrolimus (TAC), can lead to a remission rate of about 60 to 70%, but is associated with a high relapse rate and nephrotoxicity, posing a challenge for long-term treatment (3). Regimens with rituximab (RTX), which targets CD20 on B cells, are well tolerated, but only 60 to 70% of patients receiving this drug achieve sustained clinical remission. Notably, 40 to 50% of patients with persistent NS develop renal failure within 10 years, and these patients also face an increasing risk of thromboembolism and cardiovascular events (4, 5). In view of the shortcomings of traditional regimens, such as uncertain efficacy and adverse reactions, the identification of safe and effective alternative therapies for the treatment of MN has become a major challenge.

Obinutuzumab (OBZ), like RTX, is an anti-CD20 Ab, but it has higher Ab-dependent cytotoxicity, is better at depletion of B cells, and has a lower risk of immunogenicity. Thus, OBZ has emerged as an alternative for patients with MN who experience RTX treatment failure or cannot tolerate RTX (6, 7). In this paper, we describe two patients — one positive and the other negative for the PLA_2_R Ab — who had refractory MN and compared their responses to OBZ. Informed consent was obtained from each patient.

## Case reports

### Case 1

**Figure 1:**
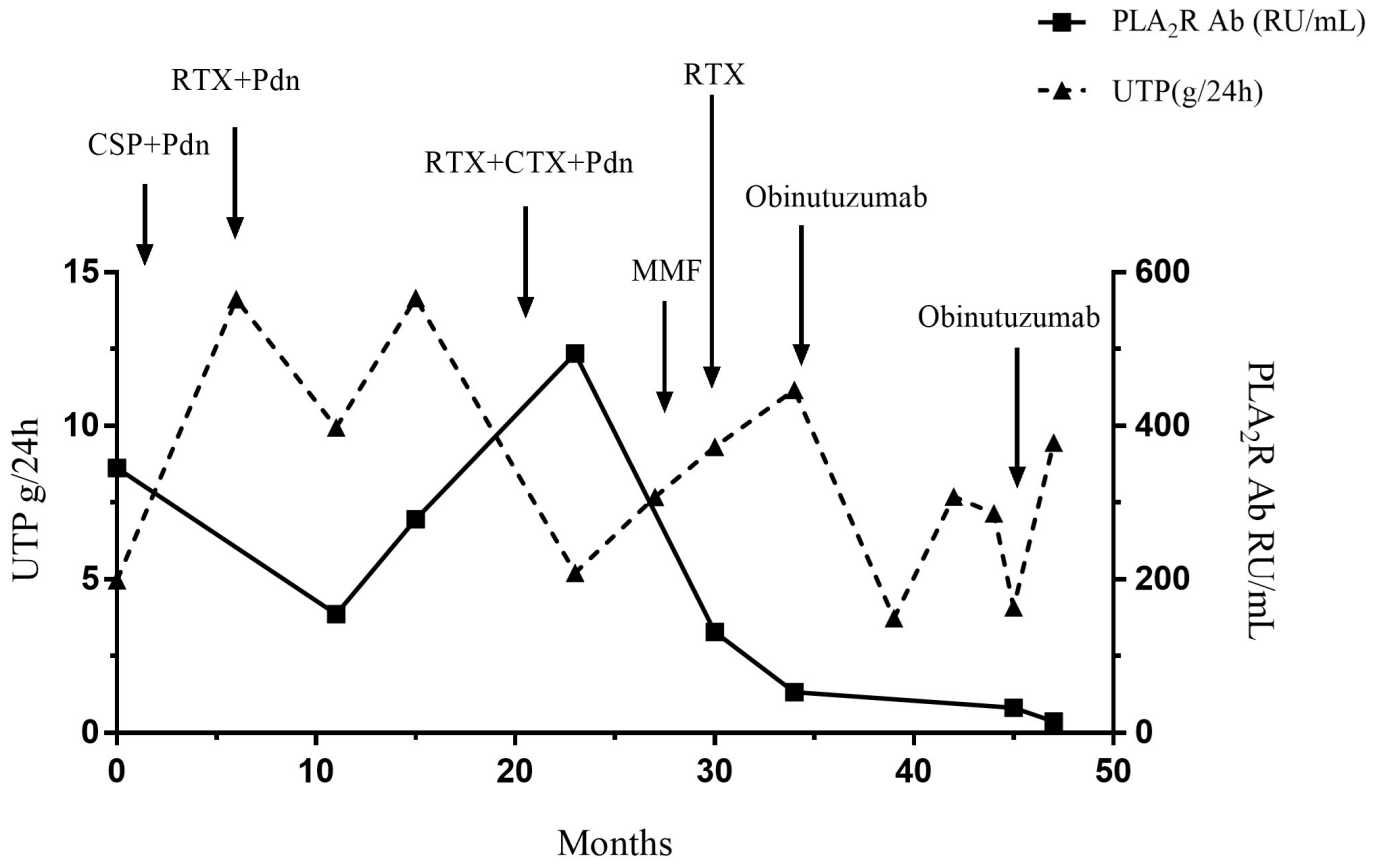
Changes of urinary protein and PLA_2_R antibody titer during treatment of Case 1.

A 50-year-old man was diagnosed with PLA_2_R-associated MN based on a kidney biopsy that was positive for the PLAR_2_R Ab after presenting with NS in September, 2019 (**Figure 1**, **Table 1**). Ultrasound data showed that the size and shape of both kidneys were normal, and the echo of both renal parenchyma was slightly enhanced. At the time of biopsy, the urine protein (UP) was 4.97 g/day, the titer of PLA_2_R Ab was 345.1 RU/mL, and he was given prednisone (20 mg qd) and CSP (100 mg bid). However, after 6 months of treatment, the UP was even higher (14.12 g/day). The prednisone dosage was maintained, but the CSP dosage was increased to 125 mg each morning and 100 mg each evening (without blood drug concentration monitoring). Five months later, the patient developed edema of the lower limbs, an increased blood pressure, UP of 9.94 g/d, serum creatinine (Scr) of 112 μmol/L, serum albumin (ALB) of 28.5 g/L, and a PLA_2_R Ab titer of 154.6 RU/mL. The patient did not respond to a variety of treatments, including RTX (cumulative dose of 6.4 g), CYC (cumulative dose of 8 g), and mycophenolate mofetil (0.75 g bid), and experienced relapse.

**Table 1:** Main treatment regimens and related laboratory indicators in Case 1 from the time of diagnosis to the last follow-up^*^.

Treatment	Dosage	Duration of Treatment	Outcome
Pdn	20 mg qd	10 months	PLA_2_R Ab 154.6 RU/mL UP 9.94 g ALB 25 g/L
CSP	100 mg bid		
Pdn	10 mg qod	4 months	PLA_2_R Ab 278.3 RU/mL UP 14.16 g ALB 28.5 g/L Scr 94 μmol/L
First course RTX	1g IV 2×		
Pdn	10 mg qod	8 months	PLA_2_R Ab 494.0 RU/mL UP 5.21 g ALB 22.8 g/L Scr 73 μmol/L
CTX	1 g IV 5×		
Pdn	10 mg qod	4 months	UP 7.68 g ALB 28.1 g/L Scr 94 μmol/L
CTX	1 g IV 3×		
Second course RTX	0.6 g IV 4×		
MMF	0.75 g bid	4 months	PLA_2_R Ab 131.4 RU/mL UP 9.31 g ALB 27 g/L Scr 84 μmol/L
Third course RTX	1 g IV 2×	4 months	PLA2R Ab 52.8 RU/mL UP 11.17 g Scr 119 μmol/L B cells (CD20) 1.79/µL
First course OBZ	1 g IV 2×	4 months	UP 3.74 g ALB 32.7 g/L
		10 months	PLA_2_R Ab 32.5 RU/mL UP 4.09 g ALB 25. 7g/L Scr 73 μmol/L B cells (CD20) 0.71/µL
		11 months	B cells (CD20) 4.41/µL
Second course OBZ	1 g IV 1×	2 months	PLA_2_R Ab 14.9 RU/mL UP 9.45 g ALB 29.2 g/L

*Pdn, prednisolone; CSP, cyclosporine A; CTX, cyclophosphamide; Scr, Serum creatinine; UP, 24 hour urinary protein; ALB, albumin; PLA_2_R Ab, phospholipase A2 receptor antibody; OBZ, obinutuzumab; MMF, mycophenolate mofetil.

We therefore administered OBZ (1 g IV on day 1, 1 g IV on day 8). Four months after this treatment, the UP decreased to 3.74 g/day and the ALB increased to 32.7 g/L. After 10 months, the patient was re-admitted due to recurrence, and the UP was 4.09 g/day, ALB was 25.7 g/L, and PLA_2_R Ab titer was 32.5 RU/mL. The patient received a second course of one-time OBZ (1 g IV). Two months later, the UP was 9.45 g/day, ALB was 29.2 g/L, and the PLA_2_R Ab titer was 14.9 RU/mL. Case 1 had no drug-related side effects. Unfortunately, at the last follow-up he reported persistent foamy urine, but declined further testing and did not return to the hospital. The patient had hypertension, gout, chronic gastritis, and a history of smoking and alcohol consumption for more than 30 years.

### Case 2

**Figure 2:**
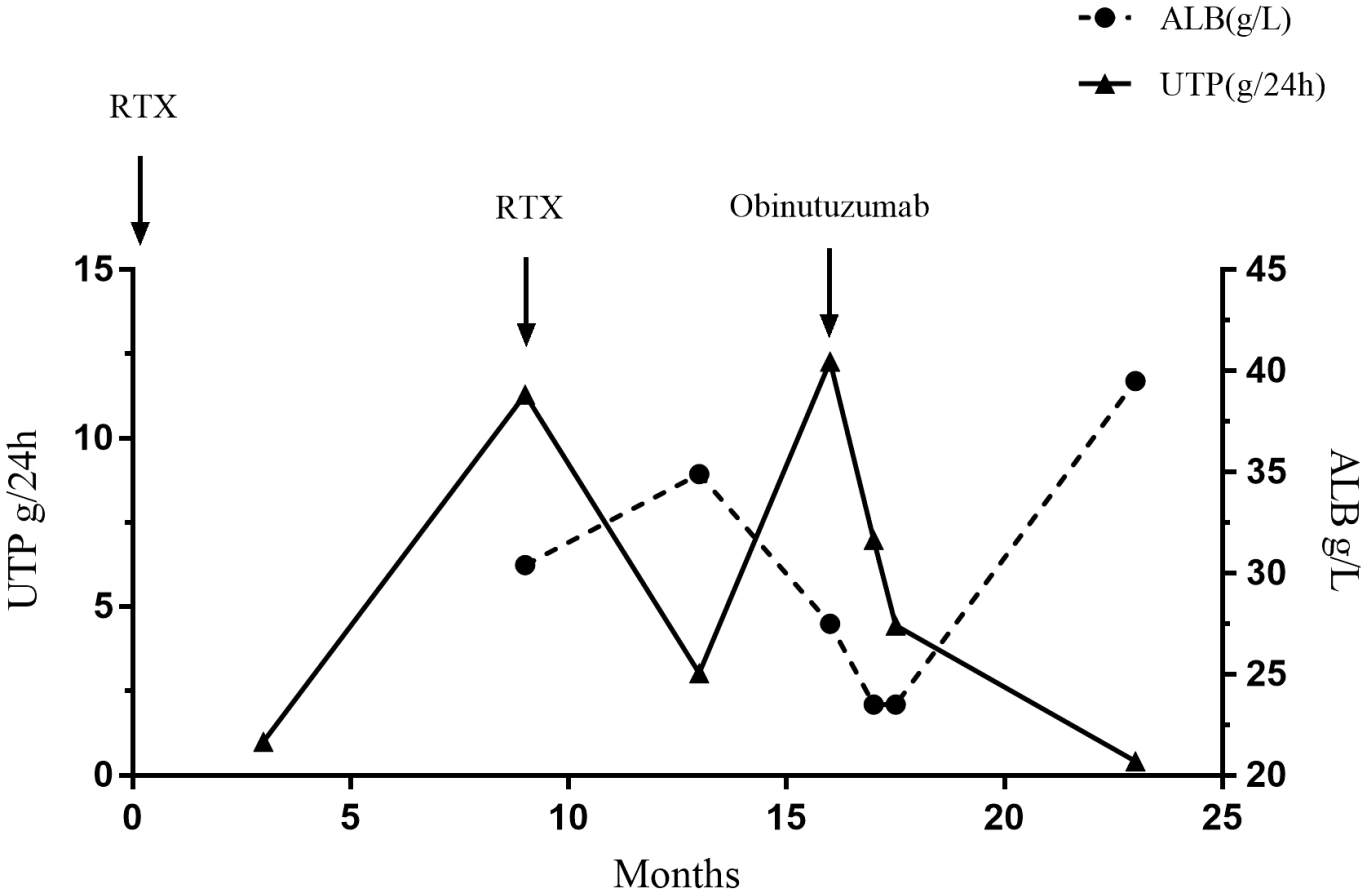
Changes of urinary protein and serum albumin during treatment of Case 2.

A 64-year-old male was admitted with foamy urine and edema, and a kidney biopsy that was positive for the PLA_2_R Ab revealed stage II membranous glomerulonephritis (MGN) in June 2017 (**Figure 2**, **Table 2**). Ultrasound data showed that the renal parenchyma was homogeneous and the corticomedullary boundary was clear in both kidneys. Calcifications and stones were found in both renal duct walls, and a small cyst was found in the left kidney. At the time of biopsy, the UP was 5.58 g and the PLA_2_R Ab titer was negative. CSP and a corticosteroid (unspecified) was the initial treatment, but CSP was discontinued due to an elevated level of Scr, so the patient was switched to TAC. However, the patient gradually developed an increased level of blood glucose, so TAC was stopped and RTX was administered (0.8 g per week for 4 weeks). Within 6 months, the UP decreased to less than 1 g/day. However, 9 months later the patient’s ALB was 30.4 g/L and the UP was 11.36 g/day. We therefore administered a second course RTX (0.8 g per week for 4 weeks). After another 4 months, the UP was 3.04 g/day and the ALB was 34.9 g/L. However, 7 months later, he experienced recurrence, with a UP of 13.85 g/day and an ALB of 29.5 g/L.

**Table 2:** Main treatment regimens and related laboratory indicators in Case 2 from the time of diagnosis to the last follow-up*.

Treatment	Dosage	Duration of Treatment	Outcome
Pdn	unknown	5 years	Drug stopped because of increased serum creatinine and blood glucose
CSA/TAC	unknown		
First course RTX	0.8 g IV 4×	3 months	UP < 1 g/day
		9 months	UP 11.3 g/d ALB 30.4 g/L Scr 88 μmol/L B cells (CD3, CD19) 2.42/µL
Second course RTX	0.8 g IV 4×	4 months	UP 3.04 g/d ALB 34.9 g/L Scr 81 μmol/L B cells (CD3, CD19) 0/µL
		7 months	UP 13.85 g/d ALB 29.5 g/L Scr 73 μmol/L B cells (CD3, CD19) 1.4/µL
OBZ	1 g IV 2×	1 month	UP 4.45 g/d ALB 23.5 g/L Scr 76 μmol/L B cells (CD3, CD19) 0/µL
		7 months	UP 0.42 g/d ALB 39.5 g/L Scr 78 μmol/L B cells (CD3, CD19) 0.04/µL
		12 months	UP<0.3 g/d

*TAC, tacrolimus; other abbreviations are in **Table 1**.

We therefore administered OBZ (1 g IV on day 1, 1g IV on day 8). A second renal biopsy that was also positive for the PLA_2_R Ab indicated stage III MN with focal scarring, which we attributed to PLA_2_R-related MN. There was also evidence of renal tubular atrophy, interstitial transformation (about 15%), and an increased glomerular volume. At one month after initiation of OBZ, the UP was 4.45 g/day. Seven months later, a reexamination showed that the ALB was 39.5 g/L, the UP was 0.42 g/day, and the Scr was 78 μmol/L, suggesting the OBZ was responsible for this near-complete clinical response. At the last follow-up (12 month later), the UP was less than 0.3g/day. Case 2 developed penile herpes after the first course of OBZ and recovered without treatment, and had a history of hypertension, sleep disorders, hepatitis B, syphilis, and a smoking history of 20 cigarettes per day for more than 40 years.

## Discussion

OBZ is a type II humanized anti-CD20 monoclonal Ab with glycosylation in the Fc region (6). This glycosylation accounts for the enhanced affinity of this drug for B cells, so it has greater cytotoxicity and stronger killing effect than RTX. OBZ was recently approved for the treatment of follicular lymphoma in China. Although no country has yet approved OBZ for treatment of MN, some evidence suggests it is useful for treatment of certain nephropathies (8–15).

In this report, each patient received at least two courses of RTX before initiation of OBZ, but these patients had different responses to OBZ. In Case 1 (PLA_2_R Ab-positive), the level of the PLA_2_R Ab titer decreased after RTX treatment, but the levels of UP and ALB did not significantly improve. After a course of OBZ (total dose of 2 g), the Scr level of this patient decreased and remained stable, the UP decreased to about 3 g/day, and the ALB level increased significantly, indicating partial remission. However, after 10 months, this patient’s UP increased again. Two months after the second course of OBZ, the PLA_2_R Ab titer in this patient decreased from 90.3 RU/mL (before treatment) to 14.9 RU/mL and remained low, but the UP was still above 9 g/day. Because the third course of RTX administration was too close to that of OBZ (less than 6 months), it is difficult to determine whether the response was attributable to RTX, OBZ, or both drugs. However, the UP remained high at the last follow-up. During the course of OBZ treatment, the level of CD20 B cells increased slightly, so the lack of a response may be related to the incomplete depletion of B-cells. However, the level of B cells remained low, although the levels before and after RTX treatment were not available. There is insufficient evidence to conclude that the decreased killing of B cells was responsible for drug resistance. Other studies examined animal tumor cells with antibody-dependent cellular cytotoxicity (ADCC) to explore the mechanism of drug resistance, and suggested a role for abnormal Fas signaling (16). Further studies are needed to establish the mechanisms of resistance to RTX and OBZ.

The response of our Case 1 was therefore similar to that of a patient in the retrospective analysis of Lin et al. (8); this other patient, who only experienced partial immune response at 3 months after treatment with OBZ, had a PLA_2_R Ab titer that decreased from 572 RU/mL to 152 RU/mL, but failed to achieve complete remission during the subsequent 13.6 months. Eleven patients in this previous study who had also had poor prior responses to RTX had significant responses to OBZ. The retrospective analysis of Hu et al. (9) also identified a patient who received OBZ and was switched to other immunosuppressants because of the deterioration of NS. However, most retrospective studies and case reports of patients with MN showed that OBZ had a better therapeutic effect than RTX (8–11).

Case 2 (PLA_2_R Ab-negative) received RTX because of the increased levels of Scr and blood glucose after CSP and TAC. This RTX treatment provided a good therapeutic effect, but the response was not long-lasting. After 6 months, the patient’s UP level increased from below 1 g/day to 11.3 g/day, and this led us to administer OBZ. One month after a course of OBZ, the UP level continued to decrease from 9 g/day to 0.42 g/day, and the ALB was 39.5 g/L at 7 months after this treatment. This patient’s level of B cells was close to 0 at 7 months after treatment, which may be the reason for the better clinical outcome than case 1.

The achievement of nearly complete clinical remission in our Case 2 (UP < 0.3 g/day, ALB ≥ 40 g/L) is consistent with the long-term remission achieved by other MN patients treated with OBZ (10). For example, Seth et al.11performed a retrospective analysis of 10 MN patients who received OBZ from January 2015 to December 2019. Seven of these patients received RTX within 1 year before initiation of OBZ; 6 of them had refractory responses, and 1 had a favorable response but switched to OBZ because of adverse effects. In addition, all patients achieved complete or partial remission at 12 months after initiation of OBZ, and the 5 patients who had longer follow-up periods-maintained remission for 24 months. OBZ has a rapid onset of action, and 60% of these MN patients had a complete or partial response (>50% decrease in UP from baseline) at 6 months. In contrast, MN patients who received RTX had a 6-month response rate of only 35%. The expression of PLA_2_R Ab does not seem to be directly related to the therapeutic effect of OBZ, because patients who were negative or positive for serum PLA_2_R Abs had better treatment outcomes. Lin et al. (8) examined patients with refractory MN who received OBZ and reported the remission was 72.2% at 6 months and 88.9% at 12 months. Notably, OBZ had a longer duration of action than RTX, so an additional dose of OBZ was unnecessary. The need for an additional course of RTX treatment may be because it cannot deplete as many B cells as OBZ. Thus, OBZ treatment typically led to an onset of remission within 6 months, and the duration of remission was longer than that provided by RTX. Our case 2 experienced complete remission within 6 months of treatment, and at 12 months later, report that UP remained below 0.3g continuously. In addition, Naik et al. (11) showed that MN patients who developed severe CKD still showed good therapeutic response to OBZ (i.e., an increase of ALB, and decreases of UP, Scr, and the PLA_2_R Abs). Similar to the report of Naik et al., each of our cases had a slight increase in the Scr level during OBZ treatment, but this level soon normalized and remained stable.

In conclusion, the results of these other studies show that OBZ, a novel humanized anti-CD20 monoclonal Ab, had good efficacy in treatment of complex refractory MN, even in patients with RTX-resistant MN, IgG4-related MN, and MN cases with severe chronic kidney disease (11–15). In addition, patients receiving OBZ had fewer hospital admissions, and OBZ is less expensive than RTX (two doses: US$2580 *vs.* US$4332). However, as illustrated in our case report, not all patients showed good therapeutic response to OBZ. However, no prospective randomized trial have yet verified that OBZ is a safe and effective first-line treatment for MN, and no studies have explicitly examined differences in efficacy between OBZ and other regimens. Nonetheless, OBZ appears to be a suitable alternative when other treatment options are ineffective or contraindicated. Further randomized controlled trials are needed to confirm whether OBZ can be widely used for treatment of patients with MN.

## Data Availability

The original contributions presented in the study are included in the article/supplementary material. Further inquiries can be directed to the corresponding author.
